# Depressive symptoms reduce when dorsolateral prefrontal cortex-precuneus connectivity normalizes after functional connectivity neurofeedback

**DOI:** 10.1038/s41598-022-05860-1

**Published:** 2022-02-16

**Authors:** Jessica Elizabeth Taylor, Takashi Yamada, Takahiko Kawashima, Yuko Kobayashi, Yujiro Yoshihara, Jun Miyata, Toshiya Murai, Mitsuo Kawato, Tomokazu Motegi

**Affiliations:** 1 Department of Decoded Neurofeedback (DecNef), Computational Neuroscience Laboratories, Advanced Telecommunications Research Institute International (ATR), Hikaridai 2-2-2. Seika-cho, Soraku, Kyoto, 619-0237 Japan; 2 Department of Cognitive, Linguistic and Psychological Sciences, Brown University, Providence, USA; 3 Medical Institute of Developmental Disabilities Research, Showa University, Tokyo, Japan; 4 Department of Psychiatry, Graduate School of Medicine, Kyoto University, Kyoto, Japan; 5 Department of Psychiatry and Neuroscience, Gunma University Graduate School of Medicine, Gunma, Japan

**Keywords:** Depression, Diagnostic markers

## Abstract

Depressive disorders contribute heavily to global disease burden; This is possibly because patients are often treated homogeneously, despite having heterogeneous symptoms with differing underlying neural mechanisms. A novel treatment that can directly influence the neural circuit relevant to an individual patient’s subset of symptoms might more precisely and thus effectively aid in the alleviation of their specific symptoms. We tested this hypothesis in a proof-of-concept study using fMRI functional connectivity neurofeedback. We targeted connectivity between the left dorsolateral prefrontal cortex/middle frontal gyrus and the left precuneus/posterior cingulate cortex, because this connection has been well-established as relating to a specific subset of depressive symptoms. Specifically, this connectivity has been shown in a data-driven manner to be less anticorrelated in patients with melancholic depression than in healthy controls. Furthermore, a posterior cingulate dominant state—which results in a loss of this anticorrelation—is expected to specifically relate to an increase in rumination symptoms such as brooding. In line with predictions, we found that, with neurofeedback training, the more a participant normalized this connectivity (restored the anticorrelation), the more related (depressive and brooding symptoms), but not unrelated (trait anxiety), symptoms were reduced. Because these results look promising, this paradigm next needs to be examined with a greater sample size and with better controls. Nonetheless, here we provide preliminary evidence for a correlation between the normalization of a neural network and a reduction in related symptoms. Showing their reproducibility, these results were found in two experiments that took place several years apart by different experimenters. Indicative of its potential clinical utility, effects of this treatment remained one-two months later.

*Clinical trial registration*: Both experiments reported here were registered clinical trials (UMIN000015249, jRCTs052180169).

## Introduction

**Figure 1:**
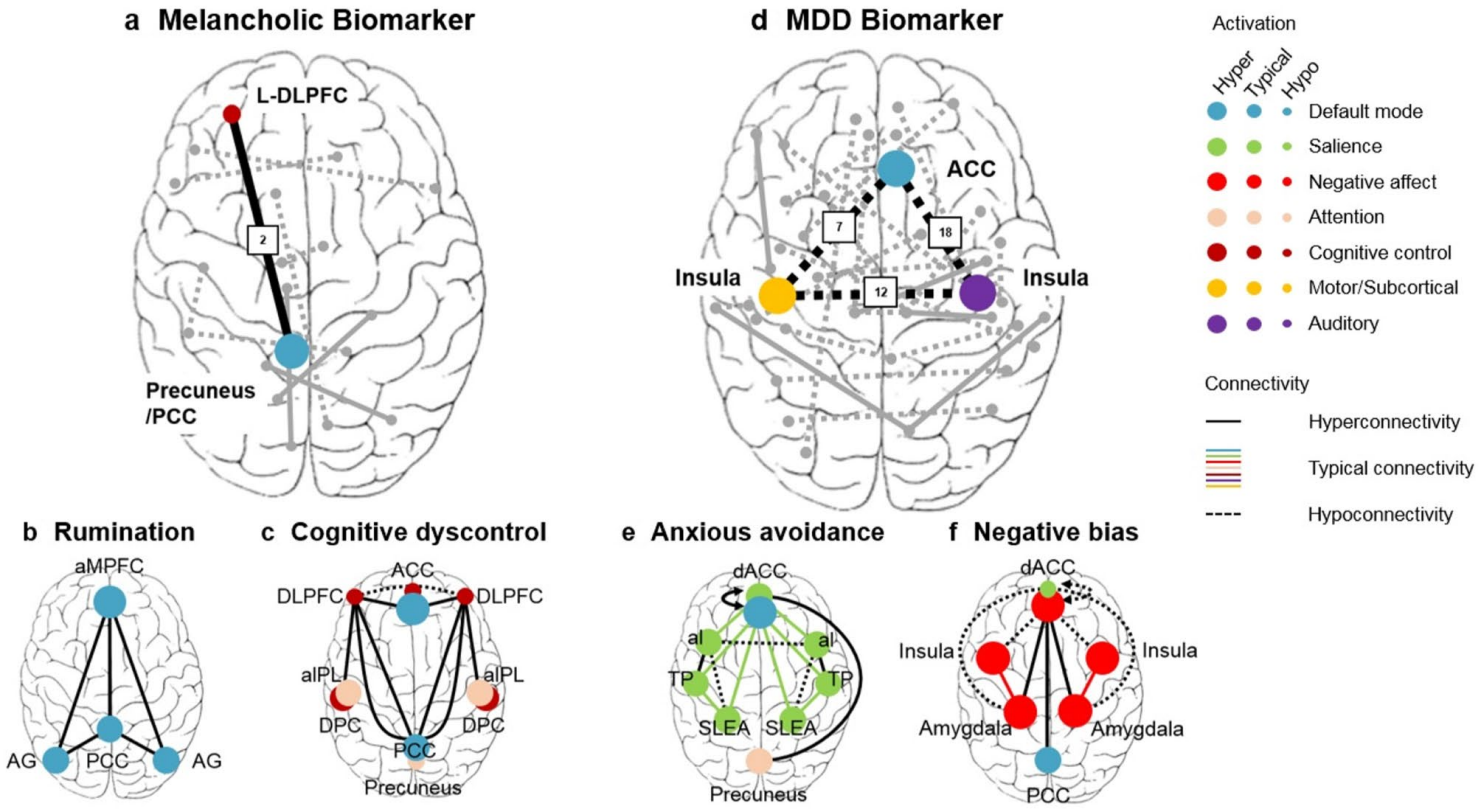
Overlap between functional connections (FCs) and regions of interest whose dysfunctions have previously been hypothesized to underlie specific depressive symptoms and those identified in data-driven biomarkers for depression (**a**) FC between the left DLPFC/mFG (from the Executive Control network) and the left precuneus/PCC (from the Default Mode network) contributed heavily to the melancholic depression biomarker of Ichikawa et al.^8^. These neural regions are also highlighted in the rumination and cognitive dyscontrol biotypes of^9^, described below. The DLPFC-PCC FC is highlighted here by the black line, while the other FCs identified in this biomarker are shown as grey lines. The number 2 indicates that this DLPFC-PCC FC had the 2nd greatest contribution to the biomarker. (**b**) The rumination biotype proposed by^9^, which involves FC disruptions within the Default Mode network that are anchored in the PCC. (**c**) The cognitive dyscontrol biotype proposed by^9^, which is characterized, in part, by hyperconnectivity between the left DLPFC and the PCC. (**d**) The MDD biomarker of^10^. The FCs identified here include hypoconnectivity between the left and right insula and hypoconnectivity between the ACC and the bilateral insula. These hypoconnectivities are also highlighted in the anxious avoidance and negative bias biotypes of^9^, described below. These hypoconnectivities are highlighted by black dotted lines. Other FCs identified in this biomarker are shown in grey. The numbers on the lines indicate the contribution of each FC to the overall biomarker (e.g. 7 indicates the FC with the 7th greatest contribution). (**e**) The anxious avoidance biotype proposed by^9^, which is, in part, characterized by hypoconnectivity between the left and right insula. (**f**) The negative bias biotype network proposed by^9^, which is, in part, characterized by hypoconnectivity between the bilateral insula and the dorsal ACC. PCC = posterior cingulate cortex (includes precuneus). aMPFC = anterior medial prefrontal cortex. AG = angular gyrus. ACC = anterior cingulate cortex. aIPL = anterior inferior parietal lobule. DPC = dorsal parietal cortex. dACC = dorsal anterior cingulate cortex. aI = anterior insula. TP = temporal pole. SLEA = sublenticular extended amygdala.

Depressive disorders contribute heavily to global disease burden^1,2^, however tolerance of their current treatments is varied, with patients still showing relatively high rates of relapse and increased mortality risk^3,4^. Different symptoms of depression have differing underlying neural mechanisms^5^; Fig. 1). This means that patients who present with different subsets of symptoms might not all benefit equally from the same treatment plan. This could explain why treatment tolerance is so variable- because relatively homogenous treatment plans are often applied uniformly to heterogeneous subsets of patients who present with different depressive symptoms. Better treatment response—for both depression and for other psychiatric disorders—may be found if diagnosis and treatment are more customized to the individual patient and the neural perturbations underlying their particular subset of symptoms^6,7^.

“Process-based neuromodulation” was proposed by Lubianiker et al.^11^ with the goal of inspiring the development of brain-targeted individualized treatments for mental dysfunctions. Specifically, Lubianker et al. proposed that mental dysfunctions should be characterized by identification of the specific underlying process(es) and neural mechanism(s). For each individual patient—the specific neural mechanism(s) related to the subset of symptoms that they present with could then be directly targeted with the goal of “normalizing” this (making it more like that of healthy controls). This could be achieved via neurofeedback, where participants are trained to modulate their own neural activity in order to influence their behavior and patterns of thinking. Indeed, it has previously been suggested that real-time functional magnetic resonance imaging (fMRI) neurofeedback may aid in the alleviation of psychiatric symptoms^12,11,13–23^. Furthermore, several proof-of-concept studies have already shown the potential effectiveness of real-time fMRI neurofeedback paradigms for depression^24–27^. Recently, a new type of real-time fMRI neurofeedback called functional connectivity neurofeedback (FCNef) has been developed^22,28–32,33^, where participants are trained to modulate functional connectivity (FC) between selected regions of interest (ROIs). This type of neurofeedback has proven effective in changing the resting-state FCs between intrinsic brain networks in the long-term^30^ and in leading to an improvement in aberrant behaviors related to neurobiological disorders^22,31,32^. Importantly, different FCs have been related to depression and its different subsets of symptoms^9,10,34–38^, see also Fig. 1). FCNef, therefore, has high potential for “process-based neuromodulation” for the treatment of depressive symptoms.

We conducted a proof-of-concept study to examine whether FCNef could be used to affect a particular subset of depressive symptoms. If a paradigm targeting one FC related to one particular subset of symptoms can efficiently and safely cause these symptoms to decrease, then in the future it would be worthwhile establishing paradigms for other subsets of symptoms as well. If this is achieved then one day FCNef might truly be of use for precision medicine, with individual patients having the specific FCs related to their own specific symptoms normalized. In this initial study, out of the different subsets of depressive symptoms, we decided to focus on rumination. This is because rumination has been well-studied in regards to underlying neural mechanisms (see below) and because rumination symptoms are easily distinguishable from other symptom subsets. Rumination is repetitive and passive self-reflection that occurs with a focus on negative emotions^39^. In short, it occurs when people repeatedly think about their feelings of sadness and the potential causes, without actively trying to resolve or fix the underlying problem^40^.

Rumination is prevalent in the melancholic subtype of depression, even though it is not generally thought to be specific to this subtype (but see^41^, who suggest that rumination is *characteristic* of melancholia). This means that the neural mechanisms underlying rumination may be observed in the brains of many patients diagnosed with the melancholic subtype. A data-driven biomarker for melancholic depression was developed by Ichikawa et al.^8^, where the fMRI resting-state data of patients diagnosed with melancholic depression was compared with that of healthy controls. Multiple FCs that are perturbed in melancholic depression were identified. Of course, not all of these are likely to relate specifically to rumination. However, the FC which was identified as having the second greatest contribution to the biomarker has previously been implicated by the literature as being related to rumination symptoms. This was the FC between the left DLPFC and left precuneus/PCC (hereby called the DLPFC-PCC FC) (Fig. 1a). The DLPFC belongs to the Executive Control network which is active during cognitively challenging tasks^42^. On the other hand, the precuneus/PCC belongs to the Default Mode network, which has baseline levels of brain activity that can be found during periods of quiet rest^43^. The Executive Control and Default Mode networks reciprocally inhibit one another^44,45^. This means that these networks alternate in activation so that only one is active at a time. Such fluctuations cause the FC between these regions to be anticorrelated in healthy people (manifesting as a negative FC). However, when ruminating, people are thought to spend an excess of time in the Default Mode network active state (see Fig. 1b). This is supported by experimental findings showing rumination symptoms to relate to a decrease in Default Mode network inhibition^46^ and particularly to hyperactivation of the precueneus/PCC^47–49^. An increase of time spent in the Default Mode network active state would result in fewer fluctuations between Default Mode network active and Executive Control network active states. This would result in people with high rumination having a reduced anticorrelation between regions from these two networks (manifesting as a less negative FC). Providing support to this idea, Bartova et al.^46^ found that—relative to healthy controls—people with remitted depression had increased Default Mode network activity during an experimental task. This resulted in a reduction in the anticorrelation between ROIs from the Default Mode and Executive Control networks, which itself related to rumination symptoms^46^. This also fits well with Ichikawa et al.’s biomarker, in which the DLPFC-PCC FC was found to have a reduced anticorrelation in patients with melancholic depression relative to healthy controls. Overall, both hypothesis-driven and data-driven evidence indicates that a reduced anticorrelation in DLPFC-PCC FC is related to rumination in depression. We therefore hypothesized that training patients to restore this anticorrelation, via FCNef, might specifically alleviate rumination symptoms.

As mentioned above, the DLPFC-PCC FC had the second greatest contribution (calculated as 0.978) to Ichikawa’s biomarker (2020). However, this was barely different from the FC with the greatest contribution in the model (calculated as 0.987) and therefore is still likely to be highly related to melancholic depression. Interestingly, Ichikawa et al.^8^ identified the DLPFC-PCC FC as uniquely failing to become normalized via treatment with SSRIs. Repetitive transcranial magnetic stimulation to one of the ROIs, the left DLPFC, has proven sometimes effective in the treatment of medication-resistant patients with depression^50^. Therefore, normalization of this particular FC with FCNef might provide treatment for depression beyond that which can be achieved using medication.

It should be noted that this is not the first study to examine whether rumination can be reduced via FCNef. A reduction of rumination symptoms after FCNef training was shown recently by Tsuchiyagaito et al.^32^. However in Tsuchiyagaito et al.’s study, a different FC was targeted: that between the precuneus and the temporoparietal junction. The selection of this FC by Tsuchiyagaito et al. was based on a connectome-wide search that was restricted to the medial PFC and PCC/precuneus and based on a priori hypotheses. Our selection of the DLPFC-precuneus FC might therefore be more objective: while selection of our FC fits well with hypotheses from the literature, our FC was also identified in a biomarker created using whole-brain data-driven analyses. Finally, Tsuchiyagaito et al.’s ROIs both come from within the Default Mode network, meaning that their paradigm should affect within-network dynamics, whereas ours, which has ROIs from both the Default Mode and the Executive Control networks, should affect between-network dynamics.

Overall, we hypothesized that normalization of the DLPFC-PCC FC should specifically relate to decreases in depressive symptoms and particularly those of brooding, which is the maladaptive form of rumination^39^. We ran a proof-of-concept study to investigate the safety and efficacy of FCNef targeting this FC. Specifically, we ran subclinical participants with depressive symptoms in a FCNef paradigm targeting this FC in two similar experiments. With the goal of restoring the anticorrelation between the DLPFC and precuneus/PCC, we rewarded our participants when this FC became more anticorrelated. Scores on the Beck Depression Inventory-II (BDI;^51^), and the Rumination Response Scale (RRS;^39,52^) (which has a brooding factor) were measured before and after FCNef training. As a control, we also measured scores on the Trait Anxiety Scale (STAI2;^53^, because anxiety has a different set of underlying neural dysfunctions^9,54^ and therefore should not be greatly affected by changes in the FC we targeted (see also Fig. 1 a–c, versus d–f, to compare different FCs and ROIs that may underlie dysfunction in more ruminative versus more anxious subtypes of depression). To further gauge the potential clinical effectiveness of this paradigm, we collected follow-up data for participants from the 2nd experiment one- and two-months after they had completed the FCNef paradigm.

## Methods

### Participants

Participants were recruited in a range of ways: we posted advertisements in local universities, on posters at train stations, on online job-seeking websites, and we even put leaflets in the mailboxes of residents of multiple of the local areas. Every person who responded and who made themselves available (between the ages of 20–40 years old) came into the laboratory to complete screening questionnaires and clinician assessment. Those who met our criteria were invited to participate in one of our main experiments. The criteria were as follows: participants must have (a) an average BDI score of over eight averaged across two BDI measurements (the range was 8.5–23.5, with a mean of 14.3 and a std of 5.1), (b) no inclination of suicidal thoughts, as measured by a question on the BDI, (c) no current or recent mental or psychiatric diseases, (d) understanding of the Japanese language. We used these criteria with the aim of recruiting people with *subclinical* levels of depression. We did not recruit participants with *high* levels of depression because we believed it was important to confirm that our paradigm did not influence our participants negatively first (of course, we expected that, if anything, our paradigm should cause positive effects, but this needed to be tested). We did not recruit *healthy* participants because we wished to avoid “floor effects”, which could come about if participants had such low levels of depressive symptoms that these could not be lowered any further. This is because such “floor effects” could potentially hide any clinical benefits of our paradigm. For these reasons, subclinical levels of depression seemed optimal for the purposes of our investigation. All participants had normal or corrected-to-normal vision. They were paid ¥8000 for each MRI session (+ a bonus in some sessions, described below) and ¥3000 for each questionnaire session (where they filled out the BDI, RRS and STAI2).

#### 1st FCNef experiment

In total, nine participants (5 males, 4 females; 23.33 ± 1.76 years old) participated in this experiment, which took place in 2016 and 2017. Specifically, these participants all completed the whole fundamental experimental procedure, which took place across 6 days and was composed of the functional localizer task and FCNef Days 0–4 (explained in detail below). The BDI and the rs-FC data (but not the RRS and STAI2 data) for seven of these participants have been reported elsewhere in a preliminary form^22^. The data for the other two participants was collected just after this previous publication by the same experimenter and so have been included in this data set.

#### 2nd FCNef experiment

**Figure 2:**
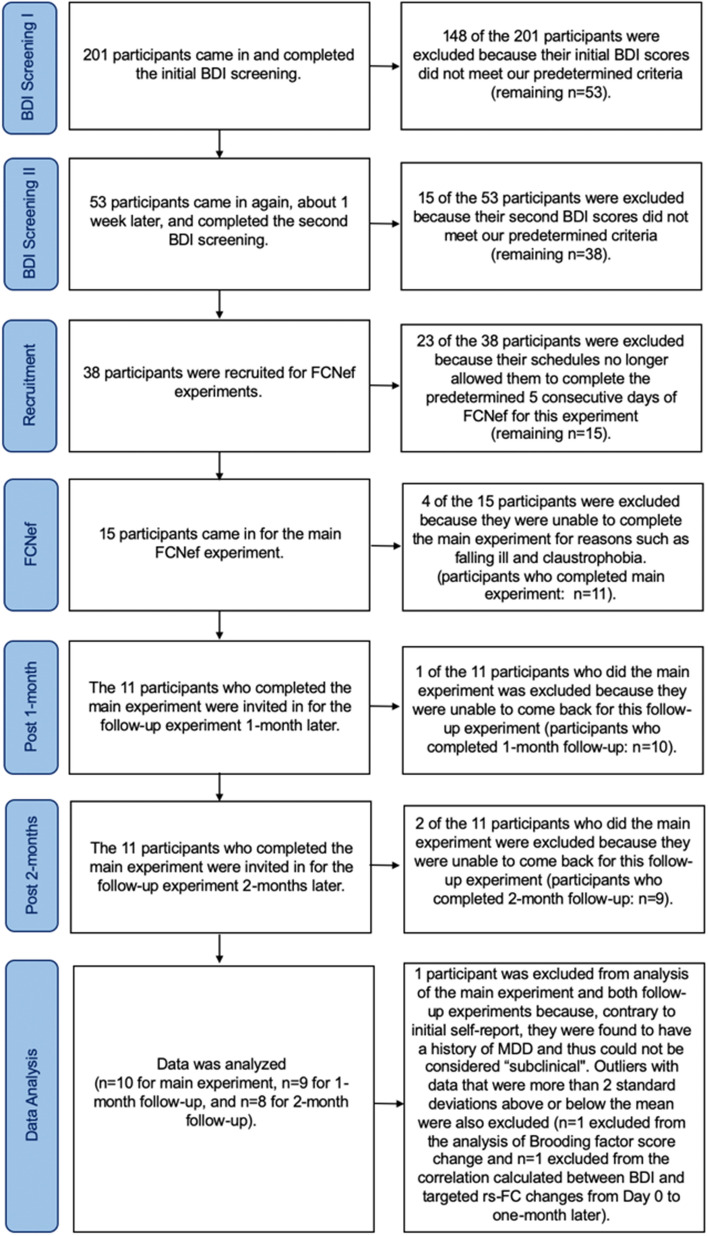
PRISMA flow diagram showing the process of participant selection for analysis for the 2nd experiment. This diagram shows how many participants were invited for and/or completed each stage of our 2nd experiment from initial screening up until data analysis. The reason for data exclusion at each stage is explained.

The design is basically the same as the 1st experiment, except for the additional examination of long-term effects. In total, 11 participants participated in this 2nd FCNef Experiment, which took place in 2019 and 2020, and was run by different experimenters from the 1st experiment. Specifically, these participants all completed the whole fundamental experimental procedure. Importantly, these participants had baseline levels of symptoms that did not differ significantly from those of the participants in the 1st experiment (see the Supplementary Results). The data from one participant was excluded, because (despite declaring no mental health problems when recruited) an in-depth interview with a psychiatrist revealed that she had just recovered from a strong case of Major Depressive Disorder (MDD). This meant that the data of 10 participants (4 males, 6 females; 23.00 ± 1.67 years old) was included in analyses. Further details on how many participants were recruited, tested, and excluded at each stage of our 2nd experiment (right up until data analysis) can be seen on Fig. 2. None of these participants had participated in the 1st FCNef experiment. Of the 11 participants who completed the 2nd experiment, nine came back for follow-up testing one-month after the main paradigm, and eight for follow-up testing two-months after the main paradigm.

### Materials

Visual stimulus presentation was controlled throughout the experiments using MATLAB 7.5.0.342 (2007b; The MathWorks Inc.). The visual stimuli were projected to an opaque screen set inside the scanner via a projector (DLA-X7-B, JVC; frame rate = 60 Hz) and a MRI compatible mirror system. Participants responded to the stimuli using response pads, which were MRI compatible (HHSC-2 × 2, Current Designs, Inc., PA, USA).

### Experimental procedure

**Figure 3:**
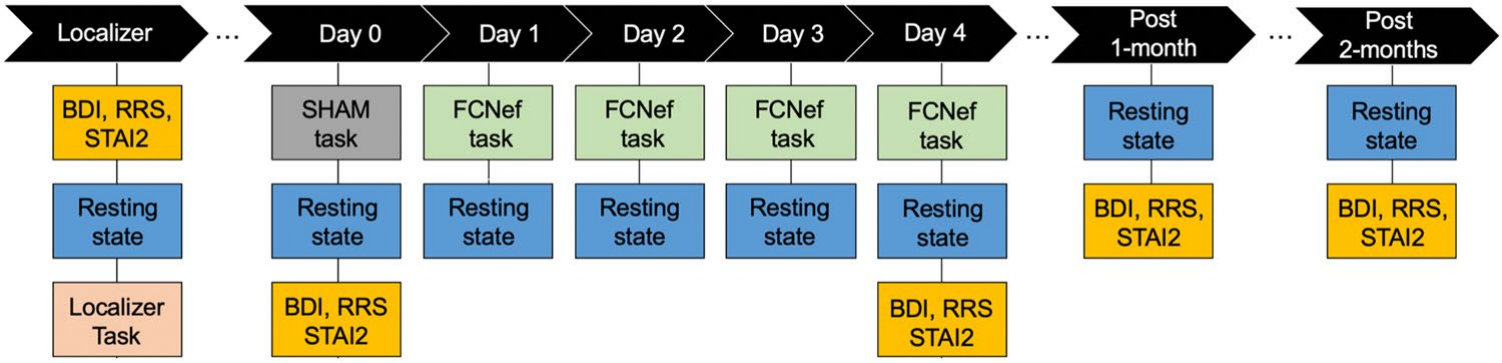
Experimental Outline. A schematic showing the general flow of experiments. Each vertical column represents a different experimental day. Periods are inserted between non-consecutive days. The post 1- and post 2-month data was only collected for the 2nd experiment. BDI = Beck’s Depression Inventory. RRS = Rumination Response Scale. STAI2 = Trait Anxiety Scale. FCNef = Functional Connectivity Neurofeedback. SHAM = SHAM FCNef.

For a general schematic of the experimental procedure, see Fig. 3. For details about the methodological rigor used in this study see the CRED-nf checklist^55^ attached in the supplementary materials (but keep in mind that ours is a proof-of-concept study, which was simply designed to test the safety and potential efficacy of our paradigm before great cost is spent to test it further).

#### Functional localizer task

Participants entered the scanner and then their resting-state fMRI was taken. Here and in all other resting-state sessions participants were simply instructed to relax and to maintain a central fixation. The resting-state scans took 10 min. After this, each participant’s T1-weighted structural MRI was taken and the localizer task subsequently began. The localizer task was the famous ‘n-back’ task, which under difficult conditions requires recruitment of the Executive Control network^56^. Use of this task therefore allowed us to identify peak DLPFC/mFG activity for each participant from times when activation of the Executive Control network was expected. Conversely, when activity from the difficult conditions of this task are subtracted from activity that occurred during rest-periods, then relative activation of the Default Mode network is expected^43^. Taking this contrast allowed us to identify peak precuneus/PCC activity for each participant.

Three sessions of the n-back task were completed by each participant. At the beginning of each session, there was first a rest-period where participants saw a black fixation cross on screen for 30 s. They had been instructed to simply relax and focus on this fixation cross. Next, in each session there were eight blocks of the n-back-task. The task rule changed from block-to-block with the order randomized within and between sessions. Each block began with written instructions which were presented on screen to inform participants of the current rule. Presentation of these instructions was followed by 10 trials in which the instructed rule should be applied. On each trial, a fixation (for 1 s) and then a number between 1 and 9, (for 2 s) was presented centrally on the screen. In the ‘0-back’ blocks (of which there were two per session), the rule was to press the response button on every trial (i.e. every time a number appeared on screen). In the ‘1-back’, ‘2-back’, and ‘3-back’ blocks (each of which there were two per session), the rule was to press the response button on the current trial if the number that appeared on screen was the same as the number that had been presented on screen one, two, or three trials beforehand, respectively. A rest-period (identical to the one at the beginning of the session) was inserted halfway through (between block 4 and 5 of) each session. Participants’ task was to follow the current rule to make as many correct responses as possible.

#### Day 0

**Figure 4:**
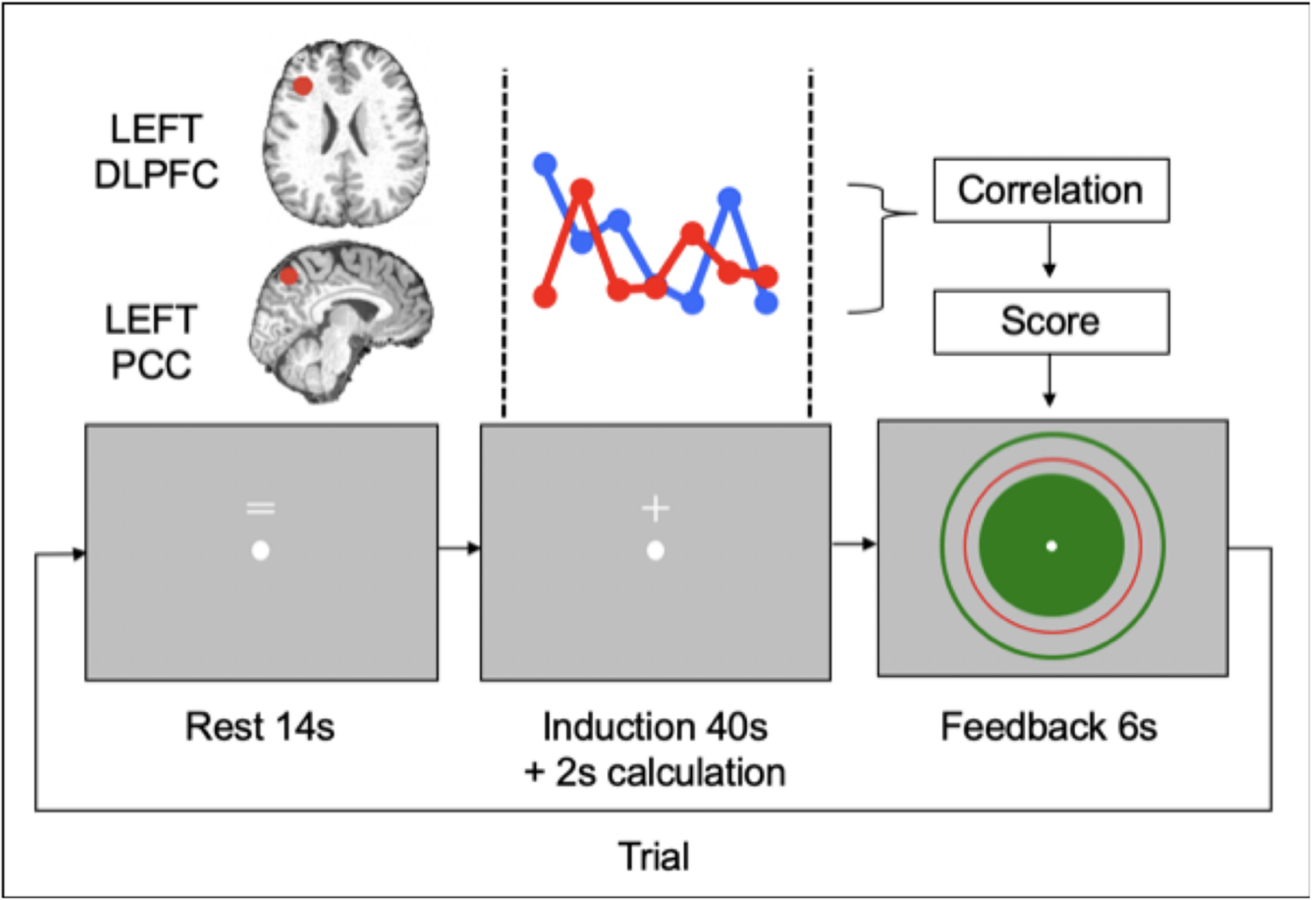
A schematic showing a trial of FCNef. On each trial, a participant saw an equals sign on the screen above the fixation point for 14 s. This was followed by a plus sign on the screen above the fixation point for 42 s. The participant had been instructed to simply maintain fixation on the fixation cross and not think about anything too deeply when the equals sign was on the screen. They had been instructed to do their best to ‘do something with their brain’ to get the best feedback possible when the plus sign was on the screen. Of the 42 s that the plus sign was on the screen, the first 40 s is considered the “induction period”. The data from the first 2 s of the induction period were discarded to somewhat account for the hemodynamic delay and the data from the next 38 s were used in the calculation of FC. Unbeknown to the participant (because nothing on the screen changed), FC calculation took place during the “calculation period”, which was the final 2 s that the plus sign was on the screen. Based on this FC calculation, feedback was then presented on the screen in the form of a green circle. The larger the green circle the more money the participant would receive for that trial. The participant had been instructed to do their best to make the green circle larger than the red circle that was also shown on the screen. The circumference of the red circle represented the participant's baseline FC (which had been calculated based on neural activity from the induction periods of SHAM FCNef from Day 0).

The purpose of the task performed on this day (called “SHAM FCNef”) was to calculate participants’ FC while they were doing the FCNef task without real feedback. This provides a baseline for each individual with which their FCs from real FCNef (performed on subsequent days) can be compared. On this day participants entered the scanner and completed five sessions of SHAM FCNef. In each session there was first a 150 s rest (of which the first 10 scans were discarded), during which participants were simply instructed to relax and focus on the onscreen fixation cross. This was followed by six trials of SHAM FCNef. What participants saw on screen during these trials was the same as what they saw on screen during the trials of real FCNef (Fig. 4), except that their baseline FC was not represented by a red circle during the feedback period.

Participants had been instructed that, during each trial while the plus sign was on screen, they should try their best to “do something with their brain” to get the best feedback possible. They were never told an explicit strategy to use to try to do this on any given trial or session, and they were never recommended to maintain or switch strategies between trials or sessions. The experimenter simply asked (between sessions) which strategy they had used. It should be noted, however, that during instructions at the beginning, a list of example strategies had been provided so that participants had a better idea of what was meant by “doing something with their brain” (see the Supplementary Materials). Participants sometimes used strategies from this list, but importantly for this experiment, none of the examples on the list were explicitly related to depression or the n-back task.

Unlike in FCNef Days 1–4, the feedback provided in SHAM FCNef was not related to the participants’ actual brain activity. Instead feedback was random. For its computation, the experimental script called upon the matlab 'rand' function and specified to use a Mersenne Twister generator for random number generation. It then used the ‘normrnd’ function to get a random number from a normal distribution with mean parameter 50 and standard deviation parameter 30.3. Participants were not aware that feedback was random—they had been instructed that the larger the feedback circle (presented onscreen during the feedback period) was in circumference, the higher their score (minimum circumference reflects a score of 0, maximum circumference reflects a score of 100). They were informed that they would receive a real cash bonus that would correspond to the sum of their scores across trials, and that they should therefore do their best to “make the circle as big as possible” on each trial. In reality, after SHAM FCNef all participants received a cash bonus of ¥500. After participants had completed SHAM FCNef, their resting-state fMRI was taken. Finally, they exited the scanner and filled out the BDI, RRS, and STAI2.

#### FCNef days 1–4

On FCNef Day 1 participants received an apology and an explanation that feedback on the previous day (Day 0) had been random, but that it would be real from the current day onwards. On all FCNef Days, participants entered the scanner and the FCNef task began. This task was completely identical to the SHAM FCNef task, except that (a) feedback (represented by a green circle) was really based on the participants’ neural activity, and (b) a red circle showing the participants baseline FC was also shown onscreen during the feedback period. Again, participants were not explicitly told to use any particular strategies on any given trial or session and no reported strategies were related to depression or the n-back task. On each trial, the DLPFC-PCC FC between the participant’s individualized ROIs was calculated online and compared to their baseline FC (see above, the Supplementary Materials, and Fig. 4 for details). The more negative the FC during the induction period (represented onscreen by the green circle) was than that individual participant’s baseline (represented onscreen by the red circle), the higher the score (up to mean—1 standard deviation where the circle reached its maximum size). By using this feedback, which corresponded to a real cash bonus (¥500 ~ ¥3000), and by not explicitly providing participants with strategies to use, the goal was to implicitly reinforce^31^ a more negative FC (more in line with that of healthy people) between these ROIs for each of the participants. After participants had completed FCNef on each day, their resting-state fMRI was taken. On FCNef Day 4 after exiting the scanner, participants filled out the BDI, RRS, and STAI2.

#### Follow-up (Post) testing (one- and two-months after FCNef)

Resting-state fMRI was taken. After this finished, participants exited the scanner and filled out the BDI, RRS, and STAI2.

### Imaging data acquisition

A 3 T scanner with a 32-channel head coil, located at the ATR Brain Activity Imaging Center, was used for scanning acquisition (Siemens MAGNETOM Verio, Siemens, Erlangen, Germany). Anatomical images were acquired using a T1-weighted MP-RAGE protocol (slice number, 240; matrix size, 256 * 256; FOV, 256 mm; voxel size, 1.0 * 1.0 * 1.0 mm (no slice gap); TR, 2300 ms; TE, 2.98 ms; flip angle, 9°). T2*-weighted images reflecting blood oxygen level-dependent (BOLD) signals were acquired in all experimental and resting state sessions using gradient-echo echo-planar imaging (EPI) (slice number, 60; matrix size, 100 * 100; FOV, 200 mm; voxel size, 2.0 * 2.0 * 2.0 mm (no slice gap); TR, 1000 ms; TE, 28 ms; flip angle, 65°). Multiband was used to allow for faster slice acquisition^57–59^. Each functional localizer task session took 590 s and consisted of 590 volumes. Each SHAM FCNef and FCNef session took 512 s and consisted of 512 volumes. Each resting state session took 600 s and consisted of 600 volumes. The first ten volumes taken in each session of all experimental and resting state sessions were discarded to ensure steady-state magnetization.

### Data analyses

“Changes” in scores and rs-FC, used in many of the analyses described below, were calculated by subtracting the data from Day 0 from the data on a later day (FCNef Day 4, one-month later, or two-months later). Outliers with data that were more than 2 standard deviations (stds) above or below the mean were excluded from data analysis. Consequently, the data from one outlier was excluded from the analysis of Brooding factor score change and the data from one outlier was excluded from the correlation calculated between BDI and targeted rs-FC changes from Day 0 to one-month later.

#### Depression, rumination, and trait anxiety scores

Depression (BDI) and trait anxiety (STAI2) scores were separately totaled for each participant individually, on every day that they were measured. Rumination scores were separately totaled for each participant for each of the three RRS factors (Depression, Brooding, and Reflection), on every day that they were measured.

#### Using the functional localizer task to make individual ROIs

The classifier for melancholic depression created by Ichikawa et al.^8^ was made based on averaged data from 130 individuals and the rs-FCs were calculated based on ROIs identified using anatomical parcellation. The results of this paper are therefore very telling in terms of overall regions of the brain that function differently for individuals with and without melancholic depression. However, ROIs defined in the same way might not be appropriate for targeting using FCNef. This is because they are larger than ROIs generally targeted in FCNef and because it is unlikely that these whole anatomically parcelled ROIs will activate fully for all individuals. Instead, smaller subsections within these larger ROIs are likely to be recruited, with the exact location of these activities differing somewhat between subjects. Indeed, this is what we found when we inspected our participants’ neural activity from the functional localizer task. Because we specifically wished to target the parts of ROIs from our target FC (DLPFC-PCC) that participants actually use, we therefore used anatomically parcelled left DLPFC/mFG and left precuneus/PCC ROIs as a guideline and identified- for each participant individually- smaller subregions within these that were active when the Executive Control and Default Mode networks, respectively, were expected to be recruited. ROIs were made for each participant based on these subregions (see Fig. 6e and f for examples). The FC between these individually identified smaller ROIs was then targeted with FCNef. Because we found overall results that look promising for our FCNef paradigm, this technique of using a data-driven biomarker to determine the general region and then using a functional localizer to determine participant-specific regions might be useful for determining target ROIs for other neurofeedback paradigms in the future.

SPM8 (Wellcome Trust Centre for Neuroimaging, University College London, UK) was used to pre-process and analyze the imaging data from the functional localizer task. Standard pre-processing steps were completed in the following order: slice-timing correction, realignment, normalization to the skull stripped T1, and spatial smoothing using a Gaussian filter (FWHM = 4 mm). Left mFG and left precuneus masks (from the Automated Anatomical Labelling (AAL) atlas^60^; were also normalized (via inverse deformation) to the skull stripped T1. A whole-brain first level factorial model was made for each participant with the activity from all three sessions of their ‘functional localizer task’ combined. Activity from the different types of blocks (0-back, 1-back, 2-back, or rest) was modelled as different conditions, which were orthogonalized. For each subject, the general linear model was used to fit the fMRI time series. Each condition was modeled from the onset until the offset of the relevant blocks. The six motion parameters were included as regressors of no interest. Once each participant’s model had been estimated, t-contrasts were estimated. Our first t-contrast was used to expose neural activity that occurs during Executive Control. The Executive Control network is expected to be more active during a task than during rest^56^, with BOLD responses increasing alongside working memory task difficulty as long as participants can correctly respond to the task^61^. This means that, as long as correct responses can be made, we would expect 3-back > 2-back > 1-back > rest activity in the Executive Control network. Our participants self-reported that they had not paid proper attention during the 3-back condition because it was too difficult and this was reflected in their behavioral responses (lower accuracy). Their neural activity from the 3-back condition was thus expected to contain a lot of noise relative to Executive Control activity and we therefore excluded it from analyses. We instead used the 2-back > rest contrast to reveal our participants’ neural activity during Executive Control. Our second t-contrast was used to expose neural activity that occurs when the Default Mode network is expected to be active. The Default Mode network is expected to be more active during rest than during a task^43^. However, in clinical patients the degree of Default Mode network activation is not always modulated by the difficulty of the task (e.g.^62^) and for this reason we included both the 1-back and the 2-back conditions in our contrast to reveal our participants’ neural activity during the default mode (the 3-back condition was excluded for the reason given above); specifically we used a rest > 1-back + 2-back contrast. For each participant, their 2-back > rest contrast was masked with their subject-space normalized left mFG mask and the peak of activation (*p* < 0.05 FWE corrected, minimum voxel size = 10) within this was determined. Likewise, for each participant their rest > 1-back + 2-back contrast was masked with their subject-space normalized left precuneus mask and the peak of activation (*p* < 0.05 FWE corrected, minimum voxel size = 10) within this was determined. Subsequently, MarsBar^63^ and the individual participants’ T1s were used to build ROIs with radiuses of 8 mm for each participant centered around these peaks. This resulted in two ROIs for each person in their own individualized brain space- one for the left mFG and one for the left precuneus. These ROIs were subsequently used as targets for each participant for calculating their feedback online during FCNef and for analysis of their related rs-FC.

#### Calculating baseline FC from SHAM FCNef data offline

Code from the FCNef toolbox (available from https://bicr.atr.jp/decnefpro/software/) and fMRI data from SHAM FCNef were first used offline to determine a baseline FC for each participant. In brief, SPM8 (Wellcome Trust Centre for Neuroimaging, University College London, UK) was used to realign and reslice volumes from the Day 0 SHAM FCNef fMRI time-series to a reference volume (which itself had been realigned to fit with the data from the functional localizer task). This data was then denoised via linear regression, with six motion parameters, a parameter for average signal over the whole brain, a parameter for average signal from cerebrospinal fluid, a parameter for average signal from grey matter, and parameters for the derivatives of all aforementioned parameters. If there were any volumes with framewise displacement > 0.5 mm then this was added as a regressor as well^64^. The data was then filtered using a Butterworth filter (with a pass band between 0.008 and 0.3 Hz). Next, correlation coefficients were calculated using the signal from the two ROIs. During this calculation, to better baseline the relevant signal from each trial, the mean signal from each ROI from the rest-period **(**at the beginning of the session) was subtracted from the signal from the same ROI from the induction period (of which signal from the first 2 s was first discarded to account somewhat for the hemodynamic delay; see also Fig. 4). Our results showed that the correlation coefficients were not normally distributed, ranging from − 1 to 1, and thus that a Fisher’s transformation was appropriate. We therefore converted the coefficients to Z-scores using the Fisher’s r-to-z transformation. The average and standard deviations of these Z-scores were determined and then these were transformed back to correlation coefficients. The resulting average correlation coefficient for each participant was used as their baseline.

#### Calculating feedback during FCNef online

For each participant, volumes from each trial of FCNef were realigned and resliced online to a reference volume (which itself had been realigned to fit with the data from the functional localizer task) using SPM8 (Wellcome Trust Centre for Neuroimaging, University College London, UK). At the end of each induction period, volumes from that trial were denoised via linear regression and filtered, with the same regressors and parameters as were used when determining the baseline. The resulting time-series from each ROI were then correlated using the same method as above. The resulting correlation coefficient was compared to the participant’s baseline FC and converted into a score (0 = baseline FC + one standard deviation or more; 50 = baseline FC; 100 = baseline FC—one standard deviation or more). This score was then used to determine the circumference of the feedback circle which was shown on screen. All of this online processing was conducted using code from the FCNef toolbox (available from https://bicr.atr.jp/decnefpro/software/).

#### Calculating DLPFC-PCC resting-state functional connectivity offline

SPM8 (Wellcome Trust Centre for Neuroimaging, University College London, UK) was used to pre-process and analyze the imaging data. Standard pre-processing steps were completed in the following order: slice-timing correction, realignment, normalization to the skull stripped T1, and spatial smoothing using a Gaussian filter (FWHM = 6 mm). This data was then denoised via linear regression, with 6 motion parameters, a parameter for average signal over the whole brain, a parameter for average signal from cerebrospinal fluid, a parameter for average signal from grey matter, and parameters for the derivatives of all aforementioned parameters. It was scrubbed so that volumes with framewise displacement > 0.5 mm were removed^64^. A temporal bandpass filter was applied to the time series using a Butterworth filter with a pass band between 0.008 and 0.1 Hz. The resulting time-series from the two ROIs were extracted and Pearson's coefficient was then calculated between them. Changes in the targeted rs-FC (from Day 0 to FCNef Day 4, or 1- or 2- months later) were correlated with changes in BDI scores, with changes in scores on the three factors of the RRS, and with changes in STAI2 scores.

### Ethics statement

This study was approved by the Ethics Committee of the Review Board of Advanced Telecommunications Research Institute International, Japan, and by the Kyoto University Certified Review Board (UMIN000015249, jRCTs052180169). All experiments were performed in accordance with relevant guidelines and regulations. All participants provided written informed consent prior to participation.

### Participant consent

All participants provided written informed consent prior to participation. Permissions: Permission to reproduce material from other sources was obtained prior to submission.

## Results

Detailed results of the two experiments separately and combined are provided in the Supplementary Materials.

### FCNef task scores, questionnaire scores, and rs-FC

Indicating successful neurofeedback training, the FCNef task scores were significantly higher on FCNef Day 4 (61.36 ± 4.91) than on FCNef Day 1 (51.18 ± 3.27) (t(18) =  − 2.31, *p* = 0.03). To further investigate if FCNef scores increased with training, a linear mixed effects model (LME) was run with a dependent variable of FCNef task score, an independent variable of FCNef Day (1, 2, 3, or 4), and an independent intercept for each participant. An ANOVA using this model showed a significant main effect of FCNef Day (see Supplementary Results). Specifically, further indicating successful neurofeedback training, the FCNef task scores increased across experimental days. This increase, as well as the relation between task scores and participants’ changes in scores on the questionnaires, can be seen on Table 1.

**Table 1:** Participants’ average scores on the FCNef task and the correlations between these task scores and before-to-after-FCNef differences in scores on the questionnaires.

	Average FCNef task scores	Corr. between task scores and BDI dif	Corr. between task scores and RRS depression factor dif	Corr. between task scores and RRS Brooding factor dif	Corr. between task scores and RRS reflection factor dif	Corr. between task scores and STAI2 dif
Day 0	52.24 ± 1.11	r = − 0.19 *p* = 0.44	r = − 0.43 *p* = 0.07	r = 0.02 *p* = 0.95	r = 0.25 *p* = 0.30	r = − 0.19 *p* = 0.45
FCNef Day 1	51.18 ± 3.27	r = − 0.01 *p* = 0.97	r = − 0.14 *p* = 0.57	r = 0.06 *p* = 0.81	r = − 0.28 *p* = 0.24	r = − 0.07 *p* = 0.30
FCNef Day 2	50.03 ± 3.91	r = 0.24 *p* = 0.32	r = 0.07 *p* = 0.77	r = 0.29 *p* = 0.23	r = 0.12 *p* = 0.61	r = − 0.04 *p* = 0.88
FCNef Day 3	59.13 ± 3.56	r = − 0.09 *p* = 0.73	r = 0.21 *p* = 0.40	r = 0.04 *p* = 0.87	r = − 0.19 *p* = 0.43	r = 0.29 *p* = 0.23
FCNef Day 4	61.36 ± 4.91	r = 0.02 *p* = 0.93	r = 0.31 *p* = 0.19	r = 0.32 *p* = 0.19	r = 0.08 *p* = 0.75	r = 0.20 *p* = 0.42

The data from both experiments are combined here. The first column shows the average and standard errors of FCNef task scores from each day of FCNef training. Each subsequent column shows the correlation statistics for correlations calculated between (a) FCNef task scores from each day, and (b) participants’ difference (“dif”) in scores on the questionnaires from before to after FCNef (FCNef Day 4—Day 0). Because the questionnaires were not taken on every day of FCNef, we were unable to correlate daily scores on the task with daily scores on the questionnaires. Task scores = scores on the FCNef task. Dif. = questionnaire scores from FCNef Day4—those from Day 0. BDI = Beck’s Depression Inventory. RRS = Rumination Response Scale. STAI2 = Trait Anxiety Scale. rs− FC = resting-state Functional Connectivity. FCNef = Functional Connectivity Neurofeedback. SHAM = SHAM FCNef.

Pre-and Post- FCNef scores on the questionnaires and DLPFC-PCC rs-FCs are shown for the 1st and 2nd experiments individually and combined in Supplementary Table 1. The specific details for correlations taken between changes in the targeted rs-FC (from Day 0 to FCNef Day 4) and changes in scores on the questionnaires are shown in Supplementary Table 2.

**Figure 5:**
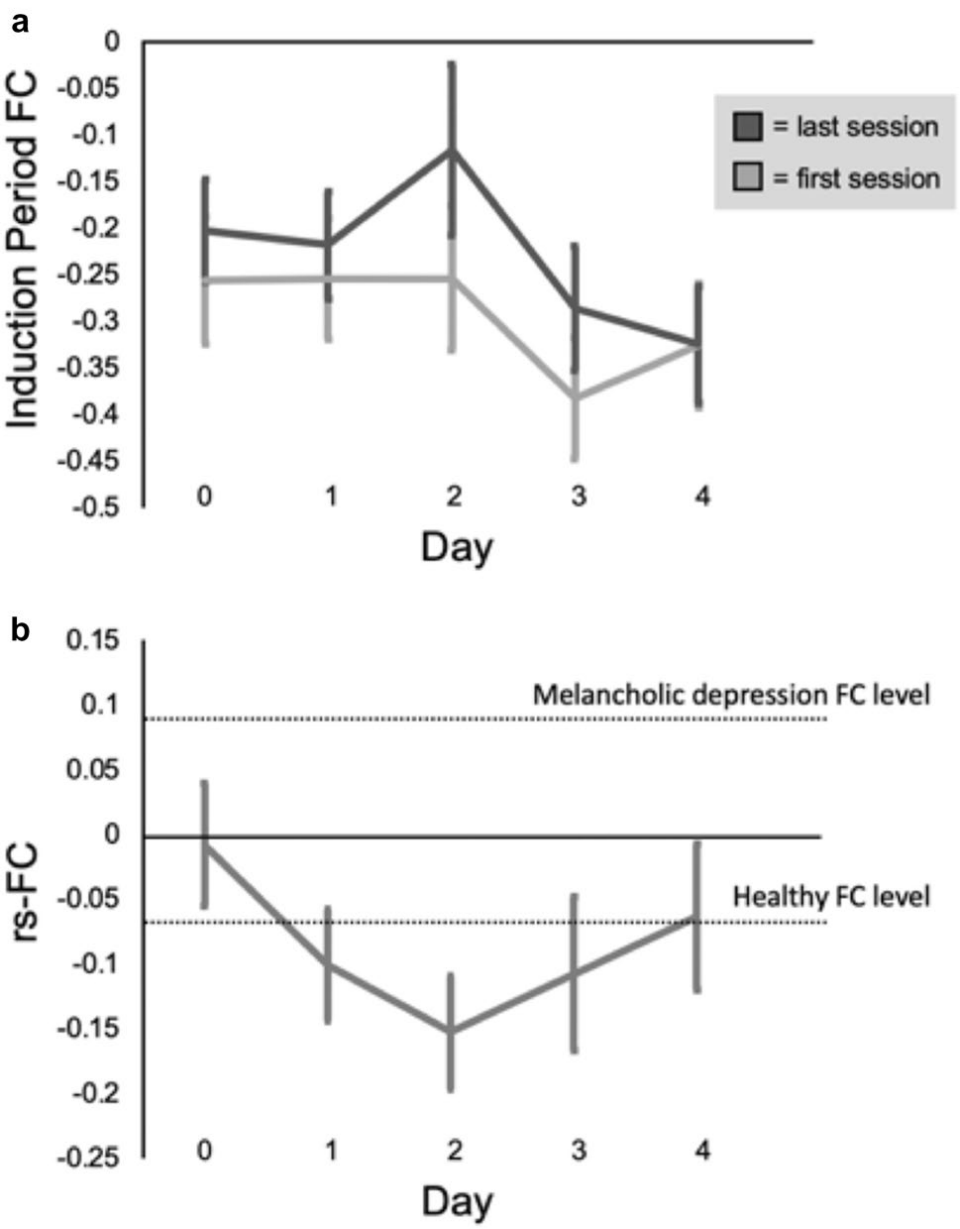
(**a**) DLPFC-PCC FC from the induction periods of the FCNef task, averaged across both experiments. Data from the first and last sessions of each day are averaged separately. Here we wish to show the data which were really used for feedback, and so this has only been put through partial preprocessing (See the Data analyses section of the Methods). This was necessary so that feedback could be displayed promptly. Participants were found to be more successful, although not significantly so, at inducing more negative anticorrelations on the first relative to the last sessions of each day. This may simply be due to an increase in fatigue, because participants often rated themselves as more sleepy on the last relative to the first session of each day. Indicating that participants learned to do the task, for both the first and the last sessions, participants were able to induce more negative anticorrelations on the final day of FCNef (FCNef Day 4) relative to on the day of SHAM FCNef (Day 0). (**b**) DLPFC-PCC FC from the resting state sessions taken on each day after neurofeedback sessions, averaged across both of our experiments. The data shown here were not used for online calculations or feedback and so have been put through the full preprocessing pipeline (see the ‘Data analyses’ section of the Methods). Full preprocessing of this data allows for better comparison of it with that from the biomarker of Ichikawa et al.^8,65^. The dotted lines represent the levels of DLPFC-PCC FC that Ichikawa et al.^8,65^ found for patients with melancholic depression and for healthy controls. As can be seen in this figure (on the grey line), our subclinical participants started out with levels of rs-FC that lay between those of Ichikawa et al.’s patients with melancholic depression and healthy controls. Across the course of FCNef training, our participants’ rs-FCs initially became even more negative than those of Ichikawa et al.’s healthy controls, and finally stabilized at the same level as those of Ichikawa et al.’s healthy controls. Vertical bars indicate the standard error of the mean.

The FCs of our subclinical patients–from the induction periods (Fig. 5a) and from the resting-state (Fig. 5b)—were more anticorrelated by the end of FCNef training (FCNef Day 4) than they were prior to FCNef training (Day 0), indicating that they became more like those of healthy controls. The rs-FC of our subclinical participants (Fig. 5b) started close to zero (0.01 ± 0.05). This lies in between the rs-FC of healthy controls from the paper of Ichikawa et al.^8^, which was negative (− 0.07 ± 0.03), and the rs-FC of patients with melancholic depression (from the same paper) which was positive (0.09 ± 0.03). As our participants with subclinical levels of depression progressed through our FCNef training paradigm, their rs-FCs became even more anticorrelated than those of healthy controls at first, and then settled somewhere close to that of healthy controls by the end (− 0.10 ± 0.04 on Day 1, − 0.15 ± 0.04 on Day 2, − 0.11 ± 0.06 on Day 3, − 0.06 ± 0.06 on Day 4). We therefore believe that our goal—of training the brains of our subclinical participants to function like those of healthy people—was achieved.

### DLPFC-PCC resting-state functional connectivity changed alongside depressive symptoms

**Figure 6:**
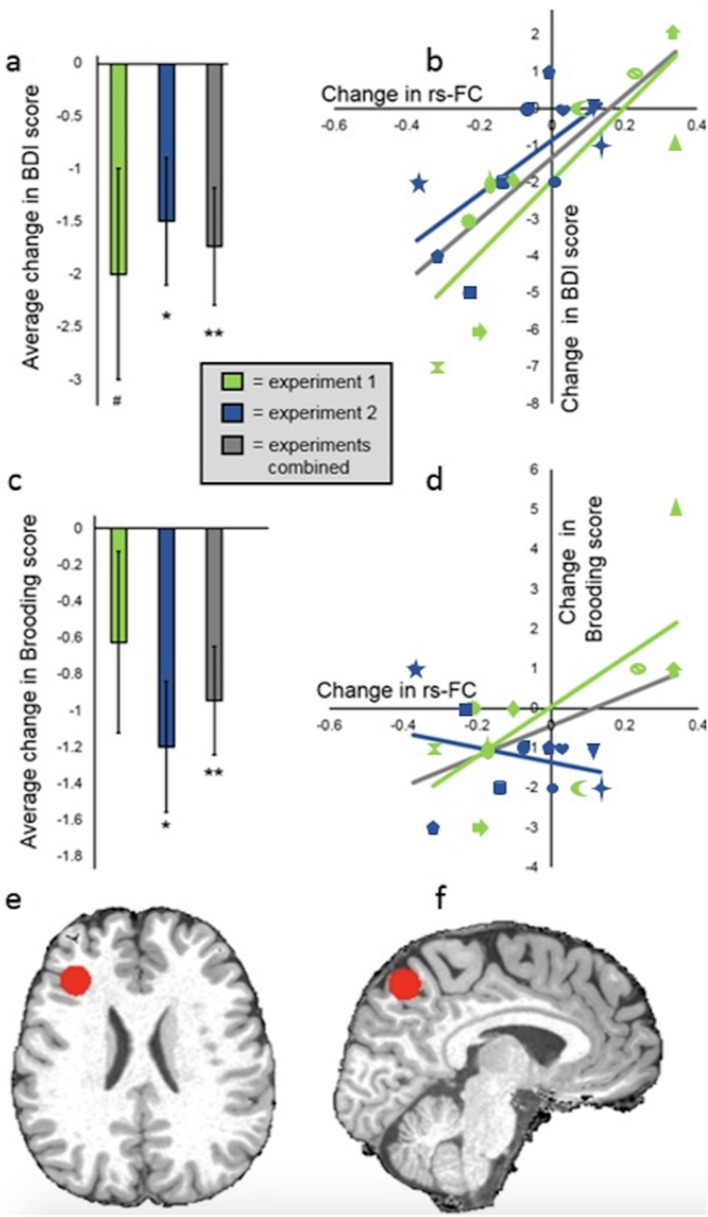
Changes that occurred in the initial period for BDI scores, RRS Brooding factor scores, and for DLPFC-PCC rs-FC. All “changes” on this figure reflect FCNef Day 4 data after Day 0 data has been subtracted away from it. The individual shapes on b. and d. represent individual participants, with the same shape being kept for each participant (**a**) Average reductions in BDI scores from Day 0 to FCNef Day 4 (for the 1st experiment t(8) =  − 2.00, *p* = 0.08; for the 2nd experiment t(9) =  − 2.50, *p* = 0.03; for the experiments combined t(18) =  − 3.12, *p* < 0.01). (**b**) Correlations between changes in BDI scores and changes in DLPFC-PCC rs-FC. In both experiments, the more this rs-FC became normalized (i.e. the stronger the anticorrelation), the greater the reduction in a participant’s BDI scores (for the 1st experiment r = 0.68, *p* < 0.05; for the 2nd experiment r = 0.67, *p* < 0.05; for the experiments combined r = 0.78, *p* < 0.001). (**c**) Average reductions in Brooding factor scores, from Day 0 to FCNef Day 4, (for the 1st experiment t(8) =  − 1.26, *p* = 0.25; for the 2nd experiment t(9) =  − 3.34, *p* < 0.01; for the experiments combined t(17) =  − 3.18, *p* < 0.01) (**d**) Correlations between changes in Brooding factor scores and changes in DLPFC-PCC rs-FC (for the 1st experiment r = 0.68, *p* =  < 0.05; note that this result was not induced by the one participant whose Brooding score drastically increased, see the Supplementary Results; for the 2nd experiment r =  − 0.28, *p* = 0.42; for the experiments combined r = 0.43, *p* = 0.06) (**e**) An example participant’s left DLPFC/MFG ROI that was made in their own subject-space is shown here, in red, rendered on their skull stripped T1. (**f**). The same example participant’s left precuneus ROI. BDI = Beck’s Depression Inventory. Brooding = Rumination Response Scale’s Brooding factor. rs-FC = resting-state Functional Connectivity. FCNef = Functional Connectivity Neurofeedback.

We ran a LME with data from the two experiments combined (LME-1). This had a dependent variable of BDI change (from Day 0 to FCNef Day 4) and an independent variable of targeted DLPFC-PCC FC rs-FC change (from Day 0 to FCNef Day 4). A likelihood ratio test showed that including a regressor for Experiment (1st or 2nd) and a regressor for its interaction with the targeted rs-FC change did not improve the model (‘BDI change ~ rs-FC change*Experiment'; AIC with the Experiment regressor and its interaction = 74.70; without them = 74.61; χ2(2) = 3.90, *p* = 0.14). This indicates that the results were not different for the two experiments. An ANOVA using the model excluding the regressors for Experiment and its interaction (‘BDI change ~ rs-FC change') showed a main effect of targeted rs-FC change (F(1,17) = 29.78, *p* < 0.001). These results indicate that change in the targeted rs-FC, from Day 0 to FCNef Day 4, was a significant predictor of change in BDI score. Regardless of whether the data of both experiments were analyzed separately or combined, a positive correlation between changes in the targeted rs-FC and BDI changes was found (Fig. 6b). These results indicate that, regardless of experiment, as the targeted rs-FC became normalized, depressive symptoms (BDI scores) were reduced. Participants’ overall reduction in depressive symptoms proved significant (Fig. 6a).

**Figure 7:**
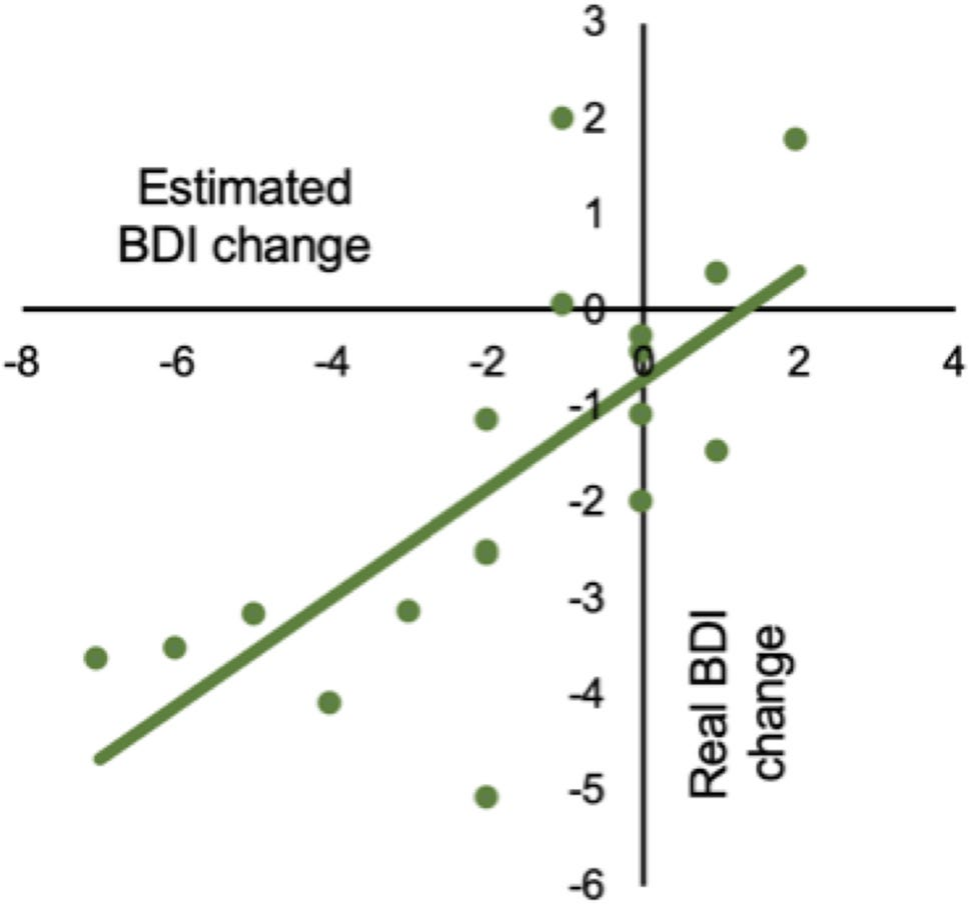
Correlations between real and estimated changes in BDI scores from before to after FCNef. LME-1 well explained participants’ changes in BDI scores from before to after FCNef using their changes in DLPFC-PCC rs-FC over the same time-period. This supports the idea that, with FCNef, changes in this rs-FC lead to changes in depressive symptoms. To ensure the results of LME-1 weren't simply driven by outliers, we next calculated the coefficients for changes in this rs-FC with each participant left out. For each participant, their changes in BDI scores were then estimated using the coefficients from the model from which they were left out. The estimated BDI changes correlated significantly and positively with real BDI changes (r = 0.70, *p* < 0.001), showing that LME-1 does not overfit and can well explain participants’ data. BDI = Beck’s Depression Inventory. rs-FC = resting-state Functional Connectivity. FCNef = Functional Connectivity Neurofeedback.

In order to exclude the possibility that the above results were obtained due to outlier participants, we excluded one participant from LME-1 and re-ran it with the same regressors. Using the estimated coefficients for the targeted rs-FC change when this participant was excluded we estimated their BDI change. We repeated this so that each participant was left-out of LME-1 once, meaning that we had estimated BDI changes for each participant. We then correlated these with their real BDI changes (Fig. 7). Estimated and real BDI changes correlated significantly and positively (r = 0.70, *p* < 0.0001). These results indicate that LME-1 was not affected by outliers and therefore provide robust support for the idea that changes in rs-FC between the targeted ROIs from before to after FCNef are predictive of changes in depressive symptoms.

**Figure 8:**
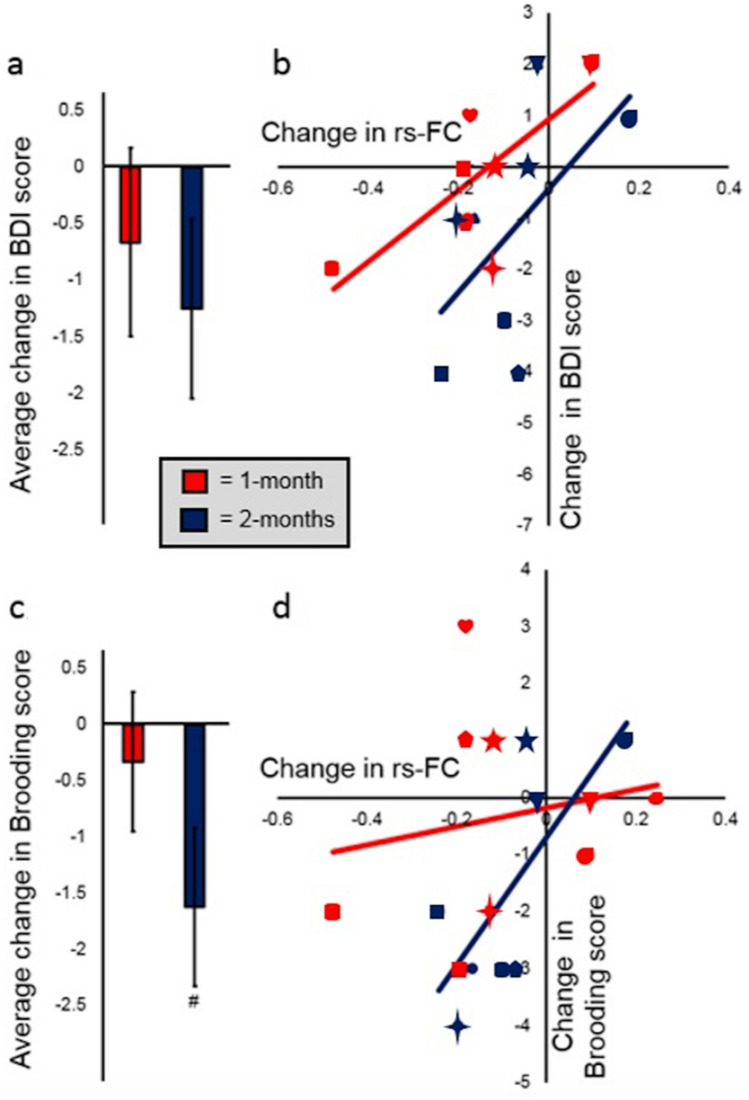
Changes that occurred in the long term for BDI scores, RRS Brooding factor scores, and for DLPFC-PCC rs-FC. Long-term data was only examined for the 2nd experiment. Changes on this figure refer either to those from Day 0 to 1-month later (data from Day 0 was subtracted from that from 1-month later; shown in red) or to those from Day 0 to 2-months later (data from Day 0 was subtracted from that from 2-months later; shown in dark blue). The individual shapes on b. and d. represent individual participants from the 2nd experiment (each participant is represented twice on each subplot because both their one- and two-month follow-up data is shown). The same shapes are kept for each participant on this figure as were displayed on Fig. 7. (**a**) Average reductions in BDI scores from Day 0 to 1- and 2-months after FCNef (for 1-month later t(8) = 0.80, *p* = 0.45; for 2-months later t(7) = 1.570, *p* = 0.16). (**b**) Correlations between changes in BDI scores and changes in the targeted rs-FC. The more this rs-FC became normalized (i.e. the stronger the anticorrelation), the greater the reduction in a participant’s BDI scores; This was significant 1-month after FCNef (r = 0.78, *p* = 0.02) and still in the same direction 2-months after FCNef (r = 0.58, *p* = 0.13). (**c**) Average reductions in Brooding factor scores from Day 0 to 1- and 2-months after FCNef (for 1-month later t(8) = 0.54, *p* = 0.61; for 2-months later t(7) = 2.30, *p* = 0.06). (**d**) Correlations between changes in Brooding factor scores and changes in the targeted rs-FC. The more this rs-FC became normalized (i.e. the stronger the anticorrelation), the greater the reduction in a participant’s Brooding factor scores. This was not significant 1-month after FCNef (r = 0.18, *p* = 0.64), but it was significant 2-months after FCNef (r = 0.73, *p* = 0.04). BDI = Beck’s Depression Inventory. Brooding = Rumination Response Scale’s Brooding factor. rs-FC = resting-state Functional Connectivity. FCNef = Functional Connectivity Neurofeedback.

In the 2nd experiment, BDI scores and rs-FC between the targeted ROIs were followed up one- and two-months after participants had completed the main paradigm (detailed information is in the Supplementary Materials). Follow-up data was not accounted for in LME-1, because it was not collected for the 1st experiment. Follow-up data for the 2nd experiment was therefore analyzed in isolation. Overall, BDI scores one- and two-months later were found to remain lower than at Day 0, but not significantly so (Fig. 8a). Correlations between changes in BDI score and changes in the targeted rs-FC remained significant one-month later (where change = data from one-month later—data from Day 0) and were maintained in a similar direction even two-months later (where change = data from two-months later—data from Day 0; Fig. 8b).

### DLPFC-PCC resting-state functional connectivity changed alongside brooding symptoms of rumination, but not alongside trait anxiety

The FC we targeted in FCNef was selected in part, because it relates to ruminative symptoms. We therefore wished to examine how changes in this rs-FC related specifically to changes in maladaptive ruminative symptoms. The RRS can be separated into three factors: Depression, Brooding, and Reflection^39^. Of these factors, the Brooding factor is specifically thought to reflect maladaptive rumination^39^. A LME similar to LME-1 was run, but with Day 0 to FCNef Day 4 changes in scores on the Brooding factor of the RRS (instead of BDI changes) as the dependent variable (LME-2; Brooding change ~ rs-FC change). A likelihood ratio test showed that including a regressor for Experiment (1st or 2nd) and a regressor for its interaction with changes in the targeted rs-FC significantly improved the model (Brooding change ~ rs-FC change*Experiment; AIC with the Experiment regressor and its interaction = 74.66; without them = 77.97; χ2(2) = 7.31 *p* = 0.03). This indicates that the results were different for the two experiments. An ANOVA using this model showed a main effect of Experiment (f(1,15) = 5.21, *p* = 0.04) that was qualified by a rs-FC change by Experiment interaction (f(1,15) = 6.43, *p* = 0.02). Correlational findings showed that, for the 1st experiment, as the targeted rs-FC became normalized, participants’ Brooding factor scores were reduced (r = 0.68, *p* < 0.05) (Fig. 6d). This finding was not replicated for the 2nd experiment (r =  − 0.28, *p* = 0.43). Overall reductions in Brooding factor scores proved significant (Fig. 6c).

In the 2nd experiment, participants’ RRS factor scores and their LPFC-PCC rs-FCs were followed up one- and two-months after participants had completed the main paradigm. Follow-up data was not accounted for in LME-2, because it was not collected for the 1st experiment. Follow-up data for the 2nd experiment was therefore analyzed in isolation. Overall, Brooding factor scores were found to remain lower than at Day 0, but only significantly so after two-months (Fig. 8c). The relationship between changes in Brooding factor scores and changes in the targeted rs-FC went from completely unrelated when data from one-month later was compared to Day 0 to being significantly related when data from two-months later was compared to Day 0 (Fig. 8d).

LMEs similar to those reported above were run, but with changes from Day 0 to FCNef Day 4 in scores on the RRS Depression factor, the RRS Reflection factor, and the STAI2 as dependent variables. No significant main effects or interactions were found in any of these LMEs. This was regardless of whether factors of Experiment and its interaction were included or excluded. When long-term effects were investigated (changes were calculated as data from one- or two-months later—data from Day 0), no significant correlations between changes in these scores and changes in the targeted rs-FC were found (See the Supplementary Materials).

## Discussion

Brooding rumination and more general depressive symptoms decreased after FCNef training targeting the DLPFC-PCC FC. While these symptoms, which are related to our targeted FC, correlated with its normalization, anxiety symptoms, that are unrelated to this FC, did not. The found effects lasted into the long-term. In the current study, due to the resource-consuming nature of the FCNef paradigm, our sample size was limited and no control neurofeedback was conducted; Therefore, caution is required when interpreting results. However, because the results of this proof-of-concept study look promising, future tests with larger sample sizes should next be conducted. Nonetheless, displaying their reproducibility and robustness; our results were found in two experiments that were carried out by different experimenters several years apart. Our FCNef paradigm therefore provides reproducible results and thus may be useful in targeted treatment of depression characterized by these particular symptoms. These results also more generally indicate the potential validity of the technique of using FCNef to target FCs from data-driven, generalizable, biomarkers for psychiatric disease and/or subsets of symptoms (see also:^66–68^).

Overall, general depressive (BDI) scores significantly decreased from before to after FCNef training (Fig. 6a). Importantly, it was found that the degree to which the targeted FC became normalized (anticorrelated) in the resting-state was a significant and robust predictor of how much a participant’s BDI scores would reduce (Figs. 6b, 7). These results indicate that our FCNef paradigm has potential clinical relevance for the treatment of depressive symptoms. The reproducibility of these findings between experiments show their robustness and clearly support the generalizability of the biomarker developed by Ichikawa et al.^8^. It was additionally found that the relationship between decreases in depressive scores and normalization of the targeted rs-FC was maintained significantly one-month later and in the same direction even two-months later (Fig. 8b). To our knowledge, this is the first study where the long-term effects of FCNef have been shown with clinical implications for depression (see also^31^ for ASD). Although more expensive than medication, these long-term effects support the practical utility of FCNef for clinical interventions^14^. Future studies may find sufficient effects with fewer days of training, because in the current study participants’ rs-FC was clearly more anticorrelated by the second day of FCNef (Fig. 5b) indicating that the full 4 days of training that we used may not have been necessary.

With potential implications for the use of FCNef in precision medicine, participants’ scores on the Brooding factor of the RRS, which are thought to reflect maladaptive rumination^39^, were significantly decreased after FCNef training (Fig. 6c). Trait anxiety symptoms, on the other hand, which are thought to be driven by different neural mechanisms^9,54^ (Fig. 1), were not found to decrease with FCNef training. This is despite the fact that the trait anxiety symptoms of our subclinical participants (like their brooding and general depressive symptoms) were initially found to be higher than those typically reported for healthy controls (see Supplementary Results and^69^). Brooding scores may have reduced here because we specifically reinforced participants when they displayed an increased anticorrelation between ROIs that belong to greater networks whose hyperconnection relates to rumination^46^ and because one of these ROIs in particular, the precuneus/PCC, has been identified as highly related to rumination^9,47–49,70^. Consistent with this, decreases in brooding symptoms (but not trait anxiety symptoms) correlated positively with normalization of the targeted FC in the resting-state (although at different times for our two experiments; this is discussed below). These results therefore have positive implications for the use of FCNef for “Process-based neuromodulation” (defined by^11^).

The correlation between changes in brooding symptoms and changes in FC was significant immediately after FCNef training for the 1st experiment (Fig. 6d) but not until two-months later for the 2nd experiment (Fig. 8d; note that these long term results were not examined for the 1st experiment). The reason for this difference in timing cannot be determined with the current data. It has been previously suggested that continued consolidation of learned neural activity might continue in the days and weeks following neurofeedback by means of Hebbian plasticity^14^. In addition or as an alternative to this, participants might continue to practice their feedback-reinforced neural activity after neurofeedback training and thereby continue to accumulate behavioural effects^14^. If these suggestions are true then effects might naturally begin to emerge at different times for different participants. Regardless of the timing at which it occurs, our results indicate that FCNef targeting DLPFC-PCC FC may have clinical benefits for the treatment of brooding symptoms.

One possibility is that ROI-based fMRI neurofeedback aimed at upregulating DLPFC activity, rather than FCNef targeting the FC between the DLPFC and precuneus/PCC, could be just as or even more effective at reducing depressive and rumination symptoms. Because the Default Mode and Executive Control networks are reciprocally inhibitory, upregulation of activity in the DLPFC (from the Executive Control network) should cause decreased activity in the precuneus/PCC (from the Default Mode network), which could aid in restoring the anticorrelation usually found between these ROIs in patients with depression. The reason we did not target upregulation of DLPFC activity is that the aforementioned reasoning is all simply hypothetical. We instead targeted the DLPFC-PCC FC itself, because not only does this fit with hypotheses from the literature but also this was identified in the melancholic biomarker of Ichikawa et al. in a data-driven manner and therefore can be considered to be objectively related to depression. A recent ROI-based fMRI neurofeedback study by Takamura et al.^25^ did, however, target upregulation of the DLPFC in patients with MDD. Successful upregulation of this activity was found to relate to a reduction in rumination, which is consistent with the hypothesis above and our own findings. However, successful upregulation of DLPFC activity was not found to relate to a reduction in general depressive (BDI) symptoms. This may mean that our paradigm using FCNef to target the DLPFC-PCC FC is more effective at reducing symptoms than a ROI-based neurofeedback paradigm that targets the DLPFC in isolation. However, considering the small sample sizes of both our own study and that of Takamura et al., this needs to be further examined.

Overall, the finding that normalization of the targeted rs-FC related to decreases in depressive (BDI) and brooding (RRS) symptoms from before to after FCNef training is consistent with the idea that FCNef caused participants’ DLPFC-PCC rs-FCs to normalize and thus their symptoms to reduce. However, our analyses were based on correlational relationships, and there was no control group or within-subject control condition (such as SHAM FCNef on all days). Therefore, it is instead possible that symptoms improved for other reasons, resulting in the found changes in this rs-FC^55,71,72^. For example, this result may have arisen due to a placebo effect. Another possibility is that our findings were contaminated by physiological noise, as was demonstrated in FCNef by Weiss et al.^73^. We did not measure respiration or heart rate in the current study and so this needs to be measured and examined in our paradigm in the future. Another possible explanation for our correlational findings is that successful self-regulation of neural activity was in itself therapeutic^74^ or that participants who were more successful at neural regulation were also more likely to try to display an improvement of symptoms (e.g. via the Hawthorne effect). However, this could not explain why symptoms that were related to the FC of interest (brooding and general depressive symptoms) decreased but other symptoms (anxiety) did not (but it is possible that successful self-regulation of neural activity simply affects different types of symptoms differently). Finally, the fact that our participants had their resting-state scans taken after the main task on each day of experimentation could mean that their resting state scans did not reflect truly intrinsic activations (see^75–77^). Nonetheless, because our main analyses involved subtracting data from Day 0 away from data from Day 4, and because the task on each of these days was identical, any task-relevant activations should have been subtracted away from the results.

Of course, although current results look promising, future study with our paradigm is still required before anything can be concluded about its causal effects. It might be of particular interest to conduct a similar FCNef experiment with an active control where another FC related to another set of symptoms is targeted. If symptoms related to the targeted FC were reduced (e.g. rumination for participants whose DLPFC-PCC FC was targeted and anxiety for participants whose bilateral insula-ACC FC was targeted; see Fig. 1), but other symptoms remained unaffected (e.g. anxiety for participants whose DLPFC-PCC FC was targeted and rumination for participants whose bilateral insula-ACC FC was targeted), then this would show causal relationships and high promise for FCNef for future precision therapy.

The results of the 1st experiment reported here are partially and briefly reported in Yamada et al.^22^. The current paper significantly extends these findings in the following ways: (1) more participants are included in the dataset of the 1st experiment; (2) a 2nd dataset, which was collected by different experimenters several years later, is additionally reported (note that, importantly, participants in the two experiments had statistically equivalent levels of baseline symptoms, see the Supplementary Results); (3) results were examined one- and two-months after the FCNef training task in the 2nd experiment, allowing for a better examination and demonstration of results in the long term; (4) additional data analyses were conducted in this paper (e.g. those regarding rumination versus anxiety) and this allows for a fuller discussion of the significance of our findings.

In addition to seven subclinical participants (whose data we included here), Yamada et al.^22^ also ran three therapy-resistant patients with MDD in their FCNef paradigm. As mentioned in the introduction, normalization of the DLPFC-PCC FC might provide treatment for depression beyond that which can be achieved using medication. Indeed, the depressive symptoms of these patients were found to dramatically reduce, which is promising but still very preliminary. In the future, now that our results have demonstrated the safety and potential efficacy of this paradigm in subclinical participants, it should be further tested in clinical trials with real patients. This is one of the main objectives of Japanese Brain/MINDS Beyond project https://brainminds-beyond.jp/ and researchers from ATR, Kyoto University, and Hiroshima University are currently working on this.

In summary, normalization of the targeted rs-FC was found to correlate with decreases in related (ruminative brooding and more general depressive), but not unrelated (trait anxiety) symptoms. These effects were found to be reproducible over two experiments and to remain for at least one-two months later, indicating that they are robust and that the FCNef paradigm may have real clinical utility. Although further testing with controls and with clinical patients is required, overall our results have proven promising for the treatment of rumination and depressive symptoms with our FCNef paradigm.

## Supplementary Information

Supplementary Information.

## Data Availability

The datasets generated during and/or analyzed during the current study are available from the corresponding author on reasonable request.
