# Supplementary material for: Depressive symptoms reduce when dorsolateral prefrontal cortex-precuneus connectivity normalizes after functional connectivity neurofeedback
Source: Sci Rep. 2022 Feb 16;12:2581. doi: 10.1038/s41598-022-05860-1 (PMC8850610; doi:10.1038/s41598-022-05860-1)
Supplement: Supplementary file 1 — Supplementary Information. [file 41598_2022_5860_MOESM1_ESM.docx]

Supplementary Materials

1. Methods

1.1. Example list of strategies

(shown to participants when they were being instructed on Day 0 and FCNef Days 1-4)

● Mathematics (addition, subtraction, multiplication, division)

● Word play (shiritori*, listing words that begin with the same first letter, listing category items such as animals, foods, or your favorite things)

● Thinking in Japanese or English

● Thinking quickly or slowly

● Continue with the same strategy across sessions or changing the strategy between sessions

● Using an easier or more difficult strategy

*Shiritori is a Japanese game where words are sequentially listed; The first letter of each word should begin with the last letter of the word before it.

2. Results

2.1. 1st FCNef Experiment

2.1.1. Results from FCNef Day 4 compared to Day 0.

For the nine participants in this experiment, average BDI scores decreased from Day 0 to the FCNef Day 4, although this only reached a trend for significance (t(8)=2.00 p=0.08). RRS scores for all three factors (Brooding, Depression, and Deflection) did not significantly change (ps>0.05) and neither did STAI2 scores (t(8)=0.48 p=0.65). The rs-FC of interest decreased from Day 0 to FCNef Day 4, although not significantly (p>0.05). These questionnaire scores and participants’ rs-FCs can be seen on Supplementary Table 1.

In support of the idea that this FC is related to depressive symptoms, a significant correlation was found between decreases in BDI and decreases in the targeted rs-FC from Day 0 to the FCNef Day 4; the more negative this rs-FC became the more participants’ depressive symptoms were reduced (r=0.68, p<0.05). In support of the idea that this FC is related to specifically Brooding symptoms, a significant correlation was found between decreases in the Brooding factor of the RRS and decreases in this rs-FC from Day 0 to FCNef Day 4; the more negative this rs-FC became the more participants’ Brooding symptoms were reduced (r=0.68, p<0.05). Note that this result was not induced just by the participant whose Brooding score dramatically increased by 5. When this person was removed from the analysis the correlation coefficient remained of a similar strength and in the same direction (r=0.58, p=0.13). Decreases in the other two factors of the RRS (Depression and Reflection) and decreases in STAI2 did not relate to decreases in this rs-FC from Day 0 to FCNef Day 4 (ps>0.05).

2.2. 2nd FCNef Experiment

2.2.1. Results from FCNef Day 4 compared to Day 0.

For the 10 participants in this experiment, BDI scores significantly decreased from Day 0 to FCNef Day 4 (t(9)=2.50, p=0.03). Scores from the Brooding factor (t(9)=3.34, p<0.01), but not the Depression or Reflection factors of the RRS (ps>0,05) were significantly decreased from Day 0 to FCNef Day 4. STAI2 scores did not significantly change (t(9)=-1.45 p=0.18). Similar to results found in the 1st FCNef experiment, the rs-FC of interest also decreased from Day 0 to FCNef Day 4, although not significantly (p>0.05). These questionnaire scores and participants’ rs-FCs can be seen on Supplementary Table 1

Interestingly, the correlation found in the 1st FCNef experiment between the decrease in BDI and the decrease in this rs-FC from Day 0 to FCNef Day 4 was replicated (r=0.67, p=0.03). The correlation found in the 1st FCNef experiment between the decrease in the Brooding factor of the RRS and the decrease in this rs-FC from Day 0 to FCNef Day 4 was not replicated for this time period (although see long-term results, below). No significant correlations between changes in scores for the other RRS factors or STAI2 and changes in the targeted rs-FC were found.

2.2.2. Long-term Results.

BDI scores, RRS scores, STAI2 scores, and DLPFC-PCC rs-FC were subsequently re-examined one-month after FCNef training for nine of the 10 participants and two-months after for eight of the 10 participants. For the nine participants who came back one-month later, BDI scores trended towards a significant reduction from Day 0 (9.89±2.12) to the FCNef Day 4 (8.40±1.83; t(8)=2.16, p=0.06), and remained lower than at Day 0, but not significantly so, one-month later (9.22±2.07; t(8)=0.80,p=0.45). Scores from the RRS Brooding factor were significantly reduced from Day 0 (9.44±1.13) to FCNef Day 4 (8.33±0.93; t(8)=2.86, p=0.02), and remained lower than at Day 0, but not significantly so, one-month later (9.11±1.18; t(8)=0.54, p=0.61). Scores on the other two RRS factors did not significantly change (ps>0.05; for Depression they went from 23.00±2.28 to 21.33±2.32 to 20.33±2.72; for Reflection they went from 9.25±1.56 to 9.33±1.76 to 8.22±1.66). Likewise, scores on the STAI2 did not significantly change from Day 0 (46.56±2.97) to FCNef Day 4 (45.00±3.72; t(9)=-1.19, p=0.27) or to one-month later (44.89±3.03; t(8)=-1.38, p=0.20). The targeted rs-FC reduced from Day 0 (0.043±0.48) to FCNef Day 4 (-0.055±0.071), but not significantly (t(8)=1.61, p=0.15), and remained lower, but not significantly so, one-month later (-0.048±0.056; t(8)=1.29, p=0.23). For the eight participants who came back 1- and two-months later, BDI scores trended towards a significant reduction from Day 0 (10.63±2.89) to FCNef Day 4 (9.00±2.35; t(7)=2.23, p=0.06), and remained lower than at Day 0, but not significantly so, one- (9.75±2.40; t(7)=0.96, p=0.37) and two-months (9.38±1.99; t(7)=1.57, p=0.16) later. Scores from the RRS Brooding factor significantly reduced from Day 0 (9.00±1.20) to FCNef Day 4 (7.88±0.99; t(7)=2.55, p=0.03). They remained lower than at Day 0, but not significantly, so one-month later (8.25±1.25; t(7)=1.43, p=0.20) and trended towards being significantly lower than at Day 0 two-months later (7.38±0.63; t(7)=2.30, p=0.06). Scores on the other two RRS factors did not significantly change (ps>0.05; for Depression they went from 21.13±2.88 to 20.50±2.45 to 19.63±2.98 to 18.63±1.65; for Reflection they went from 9.50±1.77 to 9.38±2.00 to 8.38±1.87to 8.38±1.38). STAI2 scores did not significantly decrease from Day 0 (46.25±3.35) to FCNef Day 4 (45.13±4.21; t(7)=-0.81, p=0.45), nor to one-month later (44.63±3.42; t(7)=-1.19, p=0.27), and nor to two-months later (44.00±3.36; t(7)=-1.45, p=0.19). Participants’ DLPFC-PCC rs-FCs reduced, but not significantly so, from Day 0 (-0.009±0.051) to FCNef Day 4 (-0.122±0.076; t(7)=1.70, p=0.13), and remained lower than at Day 0, but not significantly so, one-month (-0.089±0.060; t(7)=1.02, p=0.34) and two-months later (-0.090±0.035; t(7)=1.78, p=0.12).

Correlations between changes in BDI score and changes in the targeted rs-FC were significant one-month later (r=0.78, p=0.02) and were maintained in a similar direction even two-months later (r=0.58, p=0.13). The relationship between changes between Brooding factor scores from the RRS and changes in the targeted rs-FC, went from completely unrelated when data from FCNef Day 4 (r=-0.28, p=0.42) and one-month later (r=0.18, p=0.64) was compared to Day 0, to being significantly related when data from two-months later was compared to Day 0 (r=0.73, p=0.04). Changes in the other two RRS factors (Depression and Reflection) and in STAI2 scores did not significantly correlate with changes in the targeted rs-FC one- or two-months later (ps>0.05).

2.3. Experiments combined

2.3.1 Results from FCNef Day 4 compared to Day 0.

For all participants combined, BDI scores on FCNef Day 4 were found to have significantly decreased from Day 0 (t(18)=-3.12, p<0.01). Scores from the Brooding factor (t(17)=-3.18, p<0.01), but not the other RRS factors of Depression (t(18)=-0.84 p=0.41) or Reflection (t(18)=-1.02 p=0.32) were found to have significantly decreased from Day 0. STAI2 scores did not significantly change from Day 0 until FCNef Day 4 (t(18)=0.52 p=0.61). The rs-FC of interest did not significantly decrease from Day 0 to FCNef Day 4 (t(18)=-1.14 p=0.27). These questionnaire scores and participants’ rs-FCs can be seen on Supplementary Table 1.

The correlation between the decrease in BDI and the decrease in the targeted rs-FC from Day 0 to FCNef Day 4 was positive and significant (r=0.78, p<0.001). The correlation between the decrease in scores on the Brooding factor of the RRS and the decrease in this rs-FC from Day 0 to FCNef Day 4 was positive and trended towards significance (r=0.43, p=0.06). No significant correlations between changes in scores for the other RRS factors or STAI2 and changes in the targeted rs-FC were found. See Supplementary Table 2.

BDI scores were correlated with Brooding scores on Day 0 (r=0.454 p=0.05) and on FCNef Day 4 (r=0.54, p=0.02). BDI scores were also correlated with STAI2 scores on Day 0 (r=0.55, p=0.02) and on FCNef Day 4 (r=0.61, p=0.01). Furthermore Brooding scores were correlated with STAI2 scores on Day 0 (r=0.67 p<0.01) and on FCNef Day 4 (r=0.69, p<0.01). These results indicate that a participant who was likely to score more highly on one of these questionnaires was also likely to score more highly on the other two. This result is not surprising given that (a) the BDI measures a range of depressive symptoms, including those related to rumination and anxiety and (b) participants who show one set of these symptoms (e.g. Brooding) are known to be more likely to also display other related symptoms (e.g. anxiety). However, the change in BDI scores from Day 0 to FCNef Day 4 did not correlate with the change in Brooding scores (r=0.30, p=0.23); the change in BDI scores from Day 0 to FCNef Day 4 did not correlate with the change in STAI2 scores (r=-0.02, p=0.93); the change in Brooding scores from Day 0 to FCNef Day 4 did not correlate with the change in STAI2 scores (r=-0.06, p=0.79). This indicates that, despite overall relationships between symptoms measured by these three questionnaires, changes in symptomatology that occurred over the course of our experiment did not occur equally for the symptom components targeted by each of these questionnaires.

2.3.2 FCNef Task scores are predicted by experimental day

A linear mixed-effects model (LME) with data from the two experiments combined was run. This had a dependent variable of FCNef task score (mean score per day per participant) and an independent variable of Day (FCNef Days 1- 4). A likelihood ratio test showed that including an independent intercept for each subject significantly improved the model (‘Score~ Day+(1|Subject)'; AIC with the intercept regressor = 622.49; without it = 651.71; χ2(1)=31.22, p<0.001). An ANOVA using the model which included a regressor for independent intercepts showed a main effect of Day (f(1,74)=12.66 p<0.001; note that even an ANOVA using the model which excluded this regressor for independent intercepts showed a main effect of Day (f(1,74)=5.21,p=0.0250)). These results therefore indicate that the difference between scores on the first and last day was not accidental and not caused by inconsistent day-by-day fluctuation but that this was a real effect. This is supported by the data presented in the first column of Table 1, where it can be seen that the scores consistently increase day-by-day.

2.4. Comparing symptoms for participants from the two experiments

Two-sample t-tests were conducted to compare the questionnaire scores for the participants in the 1st and 2nd experiments using data from Day 0 and on Day 4 separately. T-tests calculated for data from Day 0 showed that participants from the two experiments did not differ significantly on Day 0 for their scores on any of the questionnaires: BDI for Day 0 t(17)=0.799,p=0.435; STAI2 for Day 0 t(17)=-1.358,p=0.192; RRS depression for Day 0 t(17)=-1.413,p=0.176; RRS brooding for Day 0 t(17)=-1.839,p=0.083. This indicates that participants in the two experiments did not differ in baseline levels of symptoms. T-tests calculated for data from Day 4 showed that participants from the two experiments did not differ significantly on Day 4 for their scores for BDI, RRS Depression, or STAI2: BDI for Day 4 t(17)=-0.805,p=0.432; STAI2 for Day 4 t(17)=-1.736,p=0.101; RRS depression for Day 4 t(17)=-1.728,p=0.102. These results do not therefore provide anything worthy of in-depth discussion. T-tests calculated for data from Day 4 showed that participants from the two experiments did differ significantly on Day 4 for their scores for RRS Brooding: RRS brooding for Day 4 t(17)=-2.694,p=0.015. Specifically, on Day 4, participants in the 1st experiment had significantly higher mean RRS brooding scores (mean=12.44, std=4.00) than participants in 2nd experiment (mean=8.20, std=2.82).This significant difference remained even when data from the previously identified outlier participant was excluded (the data for this participant was excluded from the analysis of Brooding factor score change described in the main manuscript because it was more than 2 stds above the mean). With this outlier removed RRS brooding for the difference between the two groups for Day 4 t(16)=-2.386,p=0.030. Even with this outlier excluded, as can be seen on Supplementary Table 1, on Day 4 participants in the 1st experiment had significantly higher mean RRS brooding scores (mean=12.13, std=4.16) than participants in the 2nd experiment (mean=8.20, std=2.82). This difference may arise from the fact that there was more variance in the Day 4 RRS Brooding score data of the 1st (std=4.00) than the 2nd (std=2.82) experiment, reflecting the finding that most participants had reduced Brooding symptoms in the 2nd experiment but the ‘change in Brooding symptoms’ was more variant in the 1st experiment (this can be seen- by proxy- by looking at the spread of data points across the top and bottom of Figure 5d, which has “changes” in Brooding scores on its y-axis). Given the small sample sizes of the two experiments and given that these experiments were essentially identical (with the only differences being the year they were conducted and the experimenters) this between-experiment difference in variance is likely to just be due to chance differences in sampling. We believe a much greater sample size would have been necessary to claim and discuss any interesting differences between the results of the two experiments.

Supplementary References

1. Brett, M., Anton, J., Valabregue, J. P. Region of interest analysis using an SPM toolbox [abstract] Presented at the 8th International Conference on Functional Mapping of the Human Brain, June 2-6, 2002, Sendai, Japan. Available on CD-ROM in NeuroImage, Vol 16, No 2.
2. Ichikawa, N. *et al*. Primary functional brain connections associated with melancholic major depressive disorder and modulation by antidepressants. *Sci. Rep*. **10**, 3542. Accessed from:<https://www.nature.com/articles/s41598-020-60527-z> doi: 10.1038/s41598-020-60527-z (2020).
3. Power, J. D., Mitra, A., Laumann, T. O., Snyder, A. Z., Schlaggar, B. L., Petersen, S. E. Methods to detect, characterize, and remove motion artifact in resting state fMRI. *NeuroImage*. **84**, 320-341 (2020).
4. Raichle, M. E. *et al*. A default mode of brain function. *Proc. Natl. Acad. Sci. USA.* **98**(2), 676-682 (2001).
5. Thompson, T. W., Waskom, M. L., Gabrieli, J. D. E. Intensive working memory training produced functional changes in large-scale frontoparietal networks. *J. Cogn. Neurosci*. **28**, 575–588 (2016).
6. Tzourio-Mazoyer, N. *et al*. Automated anatomical labeling of activations in SPM using a macroscopic anatomical parcellation of the MNI MRI single-subject brain. NeuroImage. 15:273–289 (2002).

|  | BDI Score | | RRS Brooding Score | | RRS Depression Score | | RRS Reflection Score | | STAI2 | | rs-FC | |
| --- | --- | --- | --- | --- | --- | --- | --- | --- | --- | --- | --- | --- |
|  | PRE | POST | PRE | POST | PRE | POST | PRE | POST | PRE | POST | PRE | POST |
| 1st Expt | 12.67  ±2.69 | 10.67  ±2.07 | 12.75  ±1.38 | 12.13  ±1.39 | 27.56  ±2.43 | 27.00  ±2.69 | 8.33  ±1.12 | 8.00  ±1.03 | 52.44  ±3.34 | 53.22  ±3.36 | -0.03  ±0.07 | -0.05  ±0.08 |
| 2nd Expt | 9.90  ±2.12 | 8.40  ±1.83 | 9.40  ±1.02 | 8.20  ±0.89 | 22.70  ±2.42 | 21.20  ±2.08 | 10.00  ±1.47 | 9.70  ±1.62 | 46.70  ±2.66 | 45.00  ±3.32 | 0.02  ±0.06 | -0.07  ±0.08 |
| Expts  Combined | 11.21  ±1.71 | 9.47  ±1.39 | 10.89  ±0.90 | 9.94  ±0.90 | 25.00  ±1.76 | 23.94  ±1.77 | 9.21  ±0.93 | 8.89  ±0.98 | 49.42  ±2.16 | 48.89  ±2.49 | -0.01  ±0.05 | -0.06  ±0.06 |

Supplementary Table 1. The average and standard error of participants’ rs-FC and scores on the BDI, RRS, and STAI2 prior to and after FCNef training. Pre = Day 0 (questionnaires and rs-FC were measured after the SHAM FCNef task on this day, and therefore before FCNef itself began). POST = FCNef Day 4 (questionnaires and rs-FC were measured after FCNef training on this day, and therefore after all FCNef had been completed). Expt = Experiment. BDI = Beck’s Depression Inventory. RRS = Rumination Response Scale. STAI2 = Trait Anxiety Scale. rs-FC = resting-state Functional Connectivity.

|  | | BDI | RRS Depression | RRS Brooding | RRS Reflection | STAI2 |
| --- | --- | --- | --- | --- | --- | --- |
| rs-FC | Day 0 | r=0.12  p=0.63 | r=0.27  p=0.26 | r=0.21  p=0.40 | r=0.03  p=0.90 | r=0.19  p=0.45 |
|  | FCNef  Day 4 | r=-0.30  p=0.21 | r=0.12  p=0.63 | r=0.23  p=0.34 | r=0.11  p=0.64 | r=-0.11  p=0.65 |
|  | Differences | r=0.78  p<0.001 | r=0.02  p=0.93 | r=0.43  p=0.06 | r=0.38  p=0.11 | r=0.07  p=0.78 |

Supplementary Table 2. Correlations between resting-state functional connectivity (rs-FS) and scores on the questionnaires. The data from both experiments is combined here. The first row shows correlations between the targeted rs-FC and questionnaire scores on Day 0; the second row shows correlations between the targeted rs-FC and questionnaire scores on FCNef Day 4; the third row shows correlations between differences in rs-FC (FCNef Day 4- Day 0) and differences in questionnaire scores (FCNef Day 4- Day 0). BDI = Beck’s Depression Inventory. RRS = Rumination Response Scale. STAI2 = Trait Anxiety Scale. rs-FC = resting-state Functional Connectivity. FCNef = Functional Connectivity Neurofeedback. SHAM = SHAM FCNef.
